# Biotransformation of Efavirenz and Proteomic Analysis of Cytochrome P450s and UDP-Glucuronosyltransferases in Mouse, Macaque, and Human Brain-Derived In Vitro Systems[Fn fn4]

**DOI:** 10.1124/dmd.122.001195

**Published:** 2023-04

**Authors:** Abigail M. Wheeler, Benjamin C. Orsburn, Namandjé N. Bumpus

**Affiliations:** Department of Pharmacology and Molecular Sciences, Johns Hopkins University School of Medicine, Baltimore, Maryland

## Abstract

Antiretroviral drugs such as efavirenz (EFV) are essential to combat human immunodeficiency virus (HIV) infection in the brain, but little is known about how these drugs are metabolized locally. In this study, the cytochrome P450 (P450) and UDP-glucuronosyltransferase (UGT)-dependent metabolism of EFV was probed in brain microsomes from mice, cynomolgus macaques, and humans as well as primary neural cells from C57BL/6N mice. Utilizing ultra high performance liquid chromatography high-resolution mass spectrometry (uHPLC-HRMS), the formation of 8-hydroxyefavirenz (8-OHEFV) from EFV and the glucuronidation of P450-dependent metabolites 8-OHEFV and 8,14-dihydroxyefavirenz (8,14-diOHEFV) were observed in brain microsomes from all three species. The direct glucuronidation of EFV, however, was only detected in cynomolgus macaque brain microsomes. In primary neural cells treated with EFV, microglia were the only cell type to exhibit metabolism, forming 8-OHEFV only. In cells treated with the P450-dependent metabolites of EFV, glucuronidation was detected only in cortical neurons and astrocytes, revealing that certain aspects of EFV metabolism are cell type specific. Untargeted and targeted proteomics experiments were used to identify the P450s and UGTs present in brain microsomes. Eleven P450s and 11 UGTs were detected in human brain microsomes, whereas seven P450s and 14 UGTs were identified in mouse brain microsomes and 15 P450s and four UGTs, respectively, were observed in macaque brain microsomes. This was the first time many of these enzymes have been noted in brain microsomes at the protein level. This study indicates the potential for brain metabolism to contribute to pharmacological and toxicological outcomes of EFV in the brain.

**SIGNIFICANCE STATEMENT:**

Metabolism in the brain is understudied, and the persistence of human immunodeficiency virus (HIV) infection in the brain warrants the evaluation of how antiretroviral drugs such as efavirenz are metabolized in the brain. Using brain microsomes, the metabolism of efavirenz by both cytochrome P450s (P450s) and UDP-glucuronosyltransferases (UGTs) is established. Additionally, proteomics of brain microsomes characterizes P450s and UGTs in the brain, many of which have not yet been noted in the literature at the protein level.

## Introduction

Efavirenz (EFV) is a non-nucleoside reverse transcriptase inhibitor used in combination antiretroviral therapy (cART) to treat human immunodeficiency virus (HIV)-1. Up until 2018, EFV was one component of the first-line treatment (World Health Organization, 2018) and is still used in resource-limited settings. EFV is associated with neurologic adverse events such as dizziness, depression, impaired concentration, disordered sleep, and anxiety (Fumaz et al., 2002; Gutiérrez et al., 2005; Romão et al., 2011; Ma et al., 2016; Checa et al., 2020; Hakkers et al., 2020; van Rensburg et al., 2022). Higher plasma concentrations of EFV and its primary metabolite, 8-hydroxyefavirenz (8-OHEFV), have been linked to poorer neurocognitive performance and mood changes (Grilo et al., 2016; Hakkers et al., 2020). EFV has also been shown to be toxic to neurons in culture and as it induces mitochondrial disruption (Purnell and Fox, 2014; Funes et al., 2015; Ciavatta et al., 2017). Of note, its cytochrome P450 (P450)-dependent metabolite, 8-OHEFV, has been found to elicit an even greater neurotoxic effect, demonstrating decreased neuron survival at the same dose as well as increased calcium influx and dendritic spine morphology (Tovar-y-Romo et al., 2012). Despite the negative neurologic consequences and evidence of brain penetration by EFV (Avery et al., 2013b; Thompson et al., 2015; Srinivas et al., 2019; Seneviratne et al., 2020), little is known about EFV metabolism in the brain.

Although we have highly effective combination antiretroviral therapy (cART), the brain remains a particularly elusive haven for HIV-1 infection. Vulnerable to infection (Aljawai et al., 2014; Cenker et al., 2017), the brain’s macrophages, microglia, are considered to be the primary reservoir of HIV in the brain (Ko et al., 2019). When infected by HIV-1, microglial cells release inflammatory cytokines (Walsh et al., 2014; Akiyama et al., 2020) and neurotoxic factors (van Marle et al., 2004; Eugenin et al., 2007). These contribute to increased blood-brain barrier permeability to HIV (Gandhi et al., 2010; Xu et al., 2012; Chaganti et al., 2019), prolonged neuroinflammation (Chivero et al., 2017), and cell death (van Marle et al., 2004; Eugenin et al., 2007). Individuals who are considered virally suppressed in terms of plasma HIV-1 RNA levels can have elevated HIV-1 RNA levels in the cerebrospinal fluid (CSF), which was found to correlate with adverse neurologic symptoms such as ataxia and cognitive impairment (Peluso et al., 2012). HIV-1 in the central nervous system often leads to the development of HIV-associated neurocognitive disorder (HAND), which includes symptoms such as neurocognitive impairment and functional decline (Antinori et al., 2007). Although cART has reduced the severity of HAND symptoms since the pre-cART era, the fraction of people living with HIV who develop HAND remains at approximately 42% (Wang et al., 2020). This indicates that HIV infection is persistent in the brain and underlines the importance of understanding the disposition of antiretrovirals such as EFV in the brain.

The biotransformation of EFV in the liver occurs via cytochromes P450s (P450s) and UDP-glucuronosyltransferases (UGTs). In humans, CYP2B6 and CYP2A6 form hydroxylated metabolites of EFV whereas UGT2B7 can directly glucuronidate EFV, and a variety of different UGTs can glucuronidate the P450-dependent metabolites of EFV (Mutlib et al., 1999; Ward et al., 2003; Bae et al., 2011; Ji et al., 2012; Avery et al., 2013b). P450-dependent metabolites are also noted to be sulfated, but these metabolites only account for approximately 0.7% of the excreted compound in urine and are not analyzed here (Aouri et al., 2016). In the present study, we characterize the P450 and UGT metabolism of EFV in brain using brain microsomes from mice, cynomolgus macaques, and humans as well as primary neurons, astrocytes, and microglia from C57Bl6/N mice. We show that biotransformation of EFV can occur in the brain and is carried out in a cell type–specific manner. UGT expression in brain, both in human and model species, has been historically understudied. Previous work has primarily reported on the tissue distribution of UGT mRNA (Buckley and Klaassen, 2007; Court et al., 2012; Uno and Yamazaki, 2020b), but less is currently known regarding the protein expression of UGTs in brain tissue. Therefore, a targeted mass spectrometry-based proteomics approach was developed and employed to aid in the understanding of drug metabolism in the brain by identifying the P450s and UGTs present. Several P450s and 15, 6, and 11 UGTs were detected in brain microsomes of mice, cynomolgus macaques, and humans, respectively. This work strengthens our understanding of EFV metabolism by P450s and UGTs in the brain, which will be fundamental to the investigation of the toxicity and efficacy of EFV in the brain.

## Materials and Methods

### Microsomal Metabolism Assays

Mouse and cynomolgus macaque pooled whole brain and pooled liver microsomes and pooled human brain microsomes were purchased from BioIVT (Baltimore, MD). Microsome donor pools were as follows: mouse whole brain (836), cynomolgus macaque whole brain (three), human brain (three), mouse liver (478), and cynomolgus macaque liver (five). Mouse, cynomolgus macaque, and human pooled liver microsomes were purchased from XenoTech (Kansas City, KS). Microsome donor pools were as follows: mouse liver (1000), cynomolgus macaque liver (10), and human liver (50). For the P450 metabolism assays, microsomes used at a protein concentration of 0.5 mg/ml were combined with 10 *μ*M rac EFV (Toronto Research Chemicals, Toronto, Ontario, Canada; Santa Cruz Biotechnology, Dallas, TX) and NADPH regenerating system (Corning, Corning, NY) in 80 mM potassium phosphate buffer (pH 7.4; Corning). Samples were prewarmed at 37°C for 5 minutes before initialization of reaction via the addition of microsomes. Incubations without NADPH or without substrate were used as negative controls. Reactions were incubated in a 37°C water bath with shaking for 30 minutes and quenched using equal volume ice-cold acetonitrile and stored on ice until centrifugation. For the UGT metabolism assays, brain microsomes used at a protein concentration of 0.5 mg/ml were combined with 10 *μ*M EFV, rac 8-OHEFV (Toronto Research Chemicals), or rac 8,14-diOHEFV (Toronto Research Chemicals) and a UGT reaction mixture containing 25 mM UDP glucuronic acid (UDPGA), 40 mM MgCl_2_, 250 mM Tris-HCl, and 0.125 mg/ml alamethicin (Corning). Samples were prewarmed at 37°C for 5 minutes before initialization of the reaction via the addition of microsomes. Incubations without UDPGA or substrate were used as negative controls. Reactions were incubated in a 37°C water bath with shaking for 30 minutes and quenched with equal volume ice-cold acetonitrile and stored on ice until centrifugation. Both P450 and UGT metabolism assay samples were centrifuged at 10,000 × *g* and 4°C for 5 minutes, and the supernatant was dried down in a vacufuge (Eppendorf, Hamburg, Germany). Samples were reconstituted to one-fifth the original reaction volume in 100% methanol (Optima LCMS grade; Fisher Scientific, Hampton, NH) and were separated using a HALO C18 2.7 *μ*m, 2.1 × 100 mm column (Advanced Materials Technology, Wilmington, DE) on a Dionex 3000 ultrahigh performance liquid chromatography system (uHPLC) coupled with a Q Exactive Orbitrap mass spectrometer (Thermo Fisher, Pittsburg, PA). The column heater was maintained at 30°C, and the sample injection volume was 2 *μ*l. Water with 0.1% formic acid and acetonitrile with 0.1% formic acid were used for mobile phases A and B, respectively, with a flow rate of 0.600 ml/min. From 0 to 0.5 minutes, the liquid chromatography (LC) gradient held at 25% before increasing to 85% from 0.5 to 3.5 minutes. Eighty-five percent B was held for 3.5–5.5 minutes and then decreased to 0% for 5.5–5.6 minutes. A gradient up to 25% B ran from 5.6 to 7.0 minutes and was then held for the last 0.5 minutes of the method. The Q Exactive acquired full mass spectrometry (MS) scans from 150 to 800 m/z (mass-to-charge ratio) at a resolution of 70,000 and in negative mode. The heated electrospray source ionization parameters were as follows: sheath gas flow rate = 70, auxiliary gas flow rate = 20, sweep gas flow rate = 3, spray voltage = 3.00 kV, capillary temperature = 390°C, S-lens RF level = 60.0, and auxiliary gas heater temperature = 500°C. Peak area values for efavirenz glucuronide (EFV-G) (m/z = 490.0522), 8-OHEFV (m/z = 330.0145), 8-hydroxyefavirenz glucuronide (8-OHEFV-G) (m/z = 506.0471), 8,14-dihydroxyefavirenz (8,14-diOHEFV) (m/z = 346.0099), and 8,14-diOHEFV glucuronide (8,14-diOHEFV-G) (m/z = 522.0420) were obtained in QualBrowser (Thermo Fisher) using a 5-ppm mass error tolerance.

### EFV Metabolite Formation over Time for Human Liver and Brain Microsomes

Liver and brain microsomes from pooled human donors (0.5 mg/ml) were incubated at 37°C for 0, 5, 15, 30, 60, 90, 120, 150, and 240 minutes with 100 mM potassium phosphate buffer (pH 7.4; Corning), NADPH regenerating system (Corning), UGT reaction mixture (Corning), and 5 *μ*M EFV (Toronto Research Chemicals). Samples were prewarmed at 37°C for 5 minutes before initialization of reaction via the addition of microsomes. At each incubation time point, an aliquot was removed from the reaction tube and quenched using ice-cold 100 nM EFV-d4 (Toronto Research Chemicals) in acetonitrile. Samples were stored on ice until they were centrifuged at 10,000 × *g* and 4°C for 5 minutes. The supernatant was dried in a vacufuge and reconstituted in 100% methanol. Ionized metabolites and internal standard EFV-d4 (m/z = 318.0452) were detected via the ultra high performance liquid chromatography high-resolution mass spectrometry (uHPLC-HRMS) method described above. Peak area values were obtained in QualBrowser (Thermo Fisher) using a 5-ppm mass error tolerance. Data visualization and analysis were carried out in GraphPad Prism (version 9.3.1).

### Primary Neural Cell Metabolism Assays

All cells were maintained at 37°C and 5% CO_2_. C57BL/6N cortical neurons, striatal neurons, and astrocytes were purchased from Lonza (Basel, Switzerland), and microglia were purchased from ScienCell (Carlsbad, CA). Neurons were plated at approximately 1.04 × 10^5^ cells/cm^2^ in vendor-recommended medium, and a medium exchange was performed after 2 hours. Astrocytes were plated at approximately 1.32 × 10^4^ cells/cm^2^ in vendor-recommended medium, and a medium exchange was performed after 6 hours. Microglia were plated at approximately 2.19 × 10^4^ cells/cm^2^ in vendor-recommended medium, and a media exchange was performed after 24 hours. After 3 days in culture, cells were treated with 10 *μ*M EFV, 8-OHEFV, 8,14-diOHEFV, or 0.1% DMSO vehicle control for approximately 24 hours. Cells were pelleted, and culture media was removed, dried down, and resuspended in 100% methanol. Metabolite formation was detected using a Q Exactive Orbitrap as described above.

### Proteomics Sample Preparation

Liver and brain microsomes from mice, cynomolgus macaques, and humans were prepared for proteomic analyses using S-Trap spin columns (S-Trap Micro, PN: C02-Micro-10; ProtiFi, Farmingdale, NY) according to the manufacturer’s protocol, where peptides were generated via reduction, alkylation, acidification, and tryptic digestion. Peptides were then dried down, reconstituted in water 0.1% formic acid, and quantified using a quantitative colorimetric assay (PN: 23275; Thermo Fisher) according to the manufacturer’s protocol. To improve the depth of proteins identified for the targeted P450 and UGT methods, 100 *μ*g of the brain microsome peptides were also separated into eight fractions using high pH reversed-phase chromatography (PN 84868; Thermo Fisher) according to the manufacturer’s protocol.

### Untargeted Proteomics

Peptides acquired from liver and brain microsomes (200 ng) were separated using a Waters HPLC nanoEase M/Z HSS T3, 100Å, 1.8 *μ*m, 300 *μ*m × 150 mm column and ACQUITY M Class ultra-performance liquid chromatographer (Waters, Milford, MA) before being injected onto a ZenoTOF 7600 mass spectrometer (SCIEX, Framingham, MA). Mobile phases A (water with 0.1% formic acid) and B (acetonitrile with 0.1% formic acid) flowed at 5 *μ*l/min on the following 75-minute gradient: 3%–25% B from 0 to 38 minutes, 25%–50% B from 38 to 55 minutes, and 50%–80% B from 55 to 60 minutes, and then 80% B was held for 5 minutes before decreasing back to 3% B at 67 minutes, which was held for the remaining 8 minutes. Source parameters were as follows: ion source gas 1 = 20 psi, ion source gas 2 = 60 psi, curtain gas = 35, CAD gas = 7, source temperature = 200°C, and column temperature = 30°C. TOF MS parameters were as follows: acquisition range = 400–1250 Da, accumulation time = 0.1 seconds, declustering potential = 80 V, declustering spread = 0 V, collision energy = 10 V, and collision energy spread = 0 V. TOF MSMS parameters were as follows: acquisition range = 100–1800 Da, accumulation time = 0.02 seconds, declustering potential = 80 V, declustering spread = 0 V, collision energy = 12V, collision energy spread = 5 V, maximum number of candidate enzymes = 45, intensity threshold = 150 cps. Fragmentation was achieved using collision-induced dissociation, and former candidate ions were excluded for 12 seconds after one occurrence. SCIEX .wiff files were converted to .mgf files with MS Convert before data analysis was performed in Proteome Discoverer (version 2.4.0.305; Thermo Fisher) using SEQUEST HT for peptide spectral matching with a mass error tolerance of 25 ppm. Reference proteomes obtained from UniProt for mouse (*Mus musculus*, 10090), cynomolgus macaque (*Macaca fascicularis*, 9541), and human (*Homo sapiens*, 9606) were used for protein identification.

### Targeted P450 and UGT Proteomics

Mouse, cynomolgus macaque, and human pooled liver microsomes were used to develop, validate, and optimize targeted methods for later analysis of brain microsomes. First, an untargeted proteomic analysis of the liver microsomes as described above was used to generate a list of detected peptides corresponding to P450 or UGT proteins that could be used for a targeted method. Untargeted proteomics data analysis was performed in Proteome Discoverer (version 2.4.0.305; Thermo Fisher) using SEQUEST HT for peptide spectral matching and the UniProt proteomes for mouse (*Mus musculus*, 10090), cynomolgus macaque (*Macaca fascicularis*, 9541), and human (*Homo sapiens*, 9606) were used for protein identification. Due to the relatively small number of reviewed proteins in UniProt/Swiss-Prot for cynomolgus macaque at the date of this study, an additional database was assembled from NCBI RefSeq by compiling all protein sequences available for species 9541 in NCBI Annotation Release 102 into a single fasta file. The acquired list of peptides from the untargeted proteomics was combined with the Skyline software-generated peptide prediction for 2+, 3+, and 4+ charged peptides (Skyline version 21.2.0.568; MacCoss Laboratory, University of Washington, Seattle, WA) based on protein sequences imported from UniProt (Geneva, Switzerland). A targeted proteomic analysis was performed using the same liquid chromatography (LC) gradient and MS parameters as above, first without and then with scheduled ionization based on peptide retention times. The lists of target peptides, the corresponding proteins, collision energies, and retention times can be found in Supplemental Tables 1–6. The TOF MSMS parameters for the targeted methods were as follows: acquisition range = 100–1800 Da, accumulation times = 25–80 milliseconds, declustering potential = 80 V, fragmentation = collision-induced dissociation, and collision energy varied with each individual peptide. After the target list was iteratively narrowed down to 100–200 peptides with quality peak shapes at high intensities in the liver microsomes, brain microsomes were analyzed for the presence of P450 or UGT metabolizing enzymes. Species-matched liver microsomes were run in tandem with the brain microsomes to validate findings. Targeted proteomic data analysis was performed in Skyline with the proteomes for mouse (*Mus musculus*, 10090), cynomolgus macaque (*Macaca fascicularis*, 9541), and human (*Homo sapiens*, 9606) narrowed down to just contain P450s and UGTs, ensuring consistent retention times and fragment ion distribution between identifications made in the brain microsomes and those parameters established in the species-matched liver microsomes. All samples were run in technical duplicate or triplicate and if peptide was observed in more than one brain microsome fraction, the chromatograms from fraction with the highest peak intensity were used in the figures. All proteomic files and results were deposited at the MASSIVE public repository and can be accessed at ftp://massive.ucsd.edu/MSV000090576/.

### Statistical Analysis

Data visualization and statistical analysis was carried out in GraphPad Prism (version 9.3.1; San Diego, CA). A two-tailed Welch’s *t* test was used to determine significant differences in metabolite formation between liver microsomes and brain microsomes, where *P < 0.05 and **P < 0.01.

## Results

### Brain Microsomes Demonstrate Both P450 and UGT Activity toward EFV and Its P450-Dependent Metabolites, 8-OHEFV and 8,14-diOHEFV

**Fig. 1.**
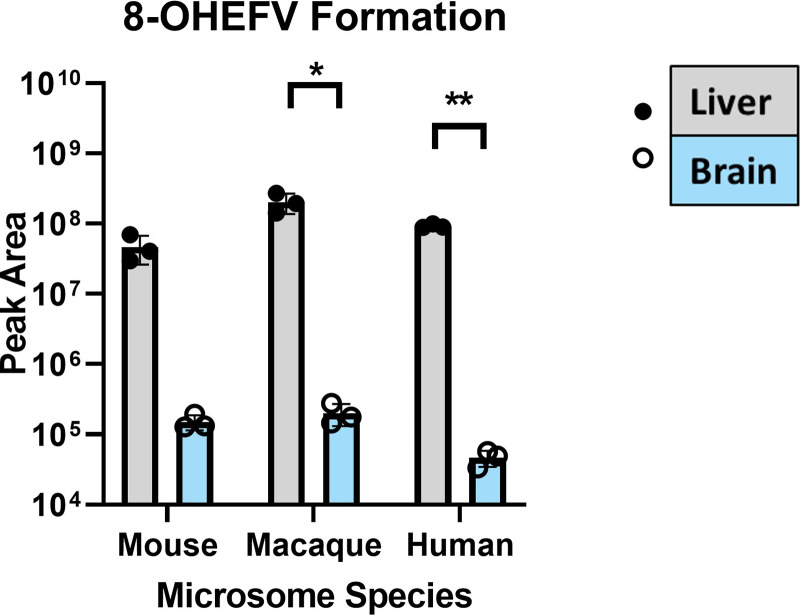
P450-dependent metabolism of EFV occurs in brain microsomes but to a lesser extent than in liver microsomes. Brain and liver microsomes (0.5 mg/ml) were incubated with 10 *μ*M EFV and NADPH cofactor for 30 minutes. The 8-OHEFV (m/z = 330.0145) metabolite was detected using uHPLC-HRMS. Formation of P450-dependent metabolites 7-OHEFV and 8,14-diOHEFV was not detected after incubation of EFV with brain microsomes from any species. Each data point represents an individual measurement from a microsomal metabolism assay. Assays were performed in triplicate. Statistical analysis of metabolite formation in liver vs. brain microsomes was performed using a two-tailed Welch’s unequal variances *t* test. *P < 0.05, **P < 0.01.

**Fig. 2.**
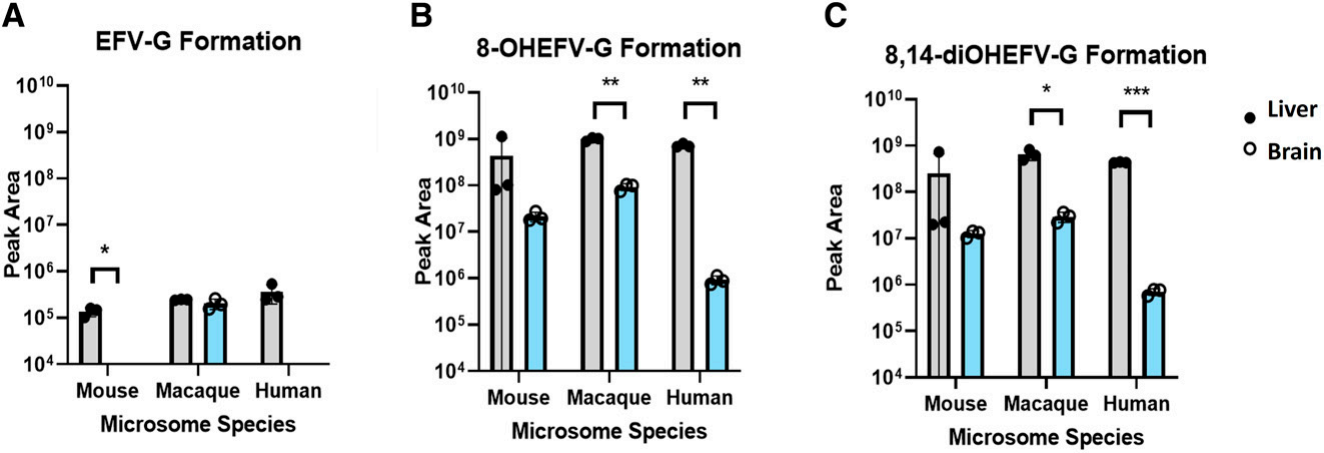
Glucuronidation of EFV is species specific, and glucuronidation of P450-dependent metabolites of EFV occurs to a greater extent in liver microsomes than in brain microsomes. Brain and liver microsomes (0.5 mg/ml) were incubated with 10 *μ*M substrate: (A) EFV, (B) 8-OHEFV, or (C) 8,14-diOHEFV, and UDPGA cofactor for 30 minutes. The metabolites EFV-G (m/z = 490.0522), 8-OHEFV-G (m/z = 506.0471), and 8,14-diOHEFV-G (m/z = 522.0420) were detected using uHPLC-HRMS. Each data point represents an individual measurement from a microsomal metabolism assay. Assays were performed in triplicate. Statistical analysis of metabolite formation in liver vs. brain microsomes was performed using a two-tailed Welch’s unequal variances *t* test. *P < 0.05, **P < 0.01, ***P < 0.001.

**Fig. 3.**
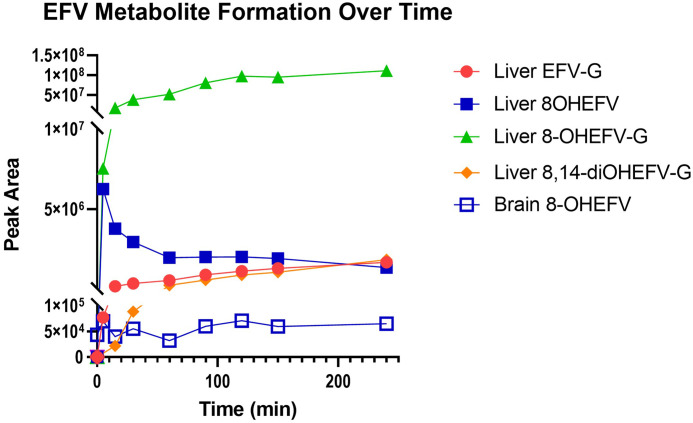
EFV metabolite formation is greater after incubation with human liver microsomes than human brain microsomes across time points. Brain and liver microsomes (0.5 mg/ml) were incubated with 5 *μ*M EFV, NADPH regenerating system, and a UGT reaction mixture (alamethicin and UDPGA cofactor) for 0, 5, 15, 30, 60, 90, 120, 150, and 240 minutes. The metabolites EFV-G (m/z = 490.0522), 8-OHEFV (m/z = 330.0145), 8-OHEFV glucuronide (8-OHEFV-G) (m/z = 506.0471), and 8,14-diOHEFV glucuronide (8,14-diOHEFV-G) (m/z = 522.0420) were detected using uHPLC-HRMS. Each data point is representative of a single measurement. Statistical analysis of total 8-OHEFV formation in liver vs. brain microsomes was performed using a two-tailed Welch’s unequal variances *t* test: *P* = 0.0033.

To characterize metabolic activity toward EFV in the brain, we employed microsomal assays utilizing pooled brain microsomes from mice, cynomolgus macaques, and humans. Brain and species-matched liver microsomes were incubated with EFV or one of its P450-dependent metabolites, 8-OHEFV or 8,14-diOHEFV. Incubation of EFV with the brain microsomes from all three species tested resulted in the formation of 8-OHEFV from EFV (Fig. 1). For each species, the formation of 8-OHEFV in the brain microsomes was less than that observed in the liver microsomes, with this difference being statistically significant for the cynomolgus macaque and human microsomes, whereas *P* = 0.0586 for the mouse microsomes. The formation of other previously reported P450-dependent EFV metabolites such as 7-OHEFV and 8,14-diOHEFV was not noted in the brain microsomes of any species tested. Metabolic assays to evaluate UGT activity toward EFV in the brain microsomes were also performed, using EFV or one of its P450-dependent metabolites as a substrate. We found the direct glucuronidation of EFV by brain microsomes to be species specific, where only the macaque brain microsomes formed EFV-G (Fig. 2A). We detected the glucuronidation of both 8-OHEFV and 8,14-diOHEFV in brain microsomes from all three species but again to a lesser extent than what we noted in the liver (Fig. 2, B and C). The differences between liver and brain microsome formation of 8-OHEFV-G and 8,14diOHEFV-G were statistically significant in the macaque and human samples. The formation of EFV metabolites in human liver and brain microsomes was then evaluated using a time course experiment. The microsomal incubations, consisting of nine timepoints over 4 hours, contained both NADPH and UDPGA cofactors to examine P450 and UGT activity concurrently. In the human brain microsomes, only 8-OHEFV was formed at a detectable level, and the peak areas observed for this metabolite were two orders of magnitude lower than the 8-OHEFV formed in the human liver microsomes. (Fig. 3). In the human liver microsomes, we observe the formation of 8-OHEFV, 8-OHEFV-G, EFV-G, and 8,14-diOHEFV-G. Additionally, the 8-OHEFV in the liver microsomes was glucuronidated at each time point investigated, where 8-OHEFV-G was the metabolite formed in the highest abundance from 15 to 240 minutes.

### Metabolism of EFV Is Cell Type Specific

**Fig. 4.**
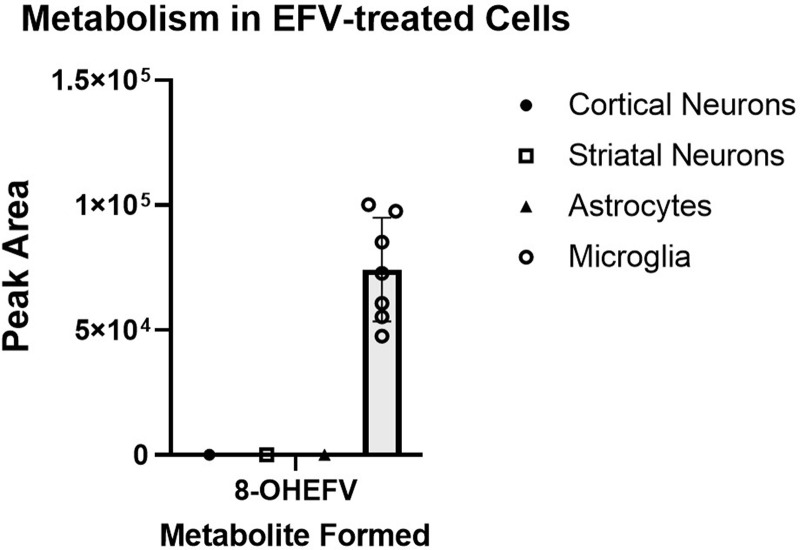
P450-dependent metabolism occurs in microglial cells from C57BL/6N mice. Cortical and striatal neurons, astrocytes, and microglia from C57BL/6N mice were incubated for 24 hours with 10 *μ*M EFV. Culture media was analyzed for metabolite formation using uHPLC-HRMS. Microglia were the only cell type tested to exhibit EFV biotransformation to 8-OHEFV. No other metabolites were observed for any of the cell types. Each data point is representative of a measurement from a single culture well. Cells were plated and treated in duplicate.

**Fig. 5.**
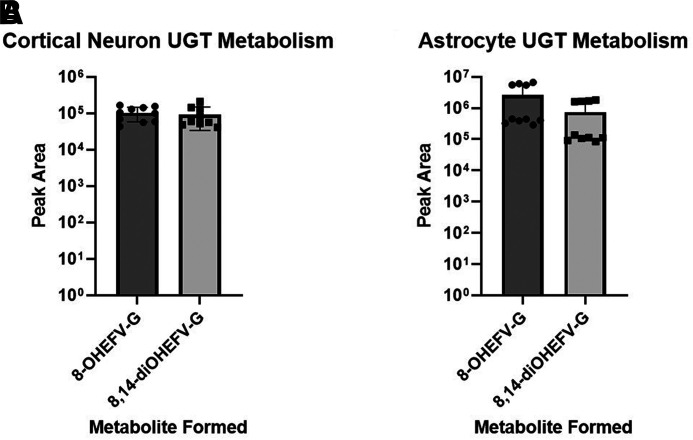
Glucuronidation of EFV P450-dependent metabolites occurs in cortical neurons and astrocytes from C57BL/6 mice. Cortical and striatal neurons, astrocytes, and microglia from C57BL/6 mice were incubated for 24 hours with 10 *μ*M 8-OHEFV or 8,14-diOHEFV. Culture media was analyzed for metabolite formation using uHPLC-HRMS. Glucuronidation of both P450-dependent metabolites was detected in the cortical neurons and astrocytes. Each data point is representative of a measurement from a single culture well. Cells were plated and treated in duplicate.

Because microsomal assays do not capture the cell type heterogeneity of the brain, EFV metabolism was studied in primary cortical and striatal neurons, astrocytes, and microglia from C57BL/6N mice. After incubation of EFV with individual cultures of each of the above-mentioned primary cell types, culture media was analyzed for the formation of EFV metabolites. The microglial cells were the only cell type where biotransformation of EFV was detected, with 8-OHEFV present in the culture medium (Fig. 4). The 8-OHEFV metabolite was the only metabolite observed. The primary cortical neurons, striatal neurons, or astrocytes did not exhibit any metabolic activity toward EFV. When synthetic P450-dependent metabolites 8-OHEFV or 8,14-diOHEFV were used as substrates, glucuronidation of both P450-dependent metabolites was detected in the cortical neurons (Fig. 5A) and astrocytes (Fig. 5B). No other P450-, UGT-, or sulfotransferase-dependent metabolites were observed.

### Untargeted Proteomics Confirmed Higher Abundance of P450s and UGTs in the Liver Microsomes Compared with the Brain Microsomes

**Fig. 6.**
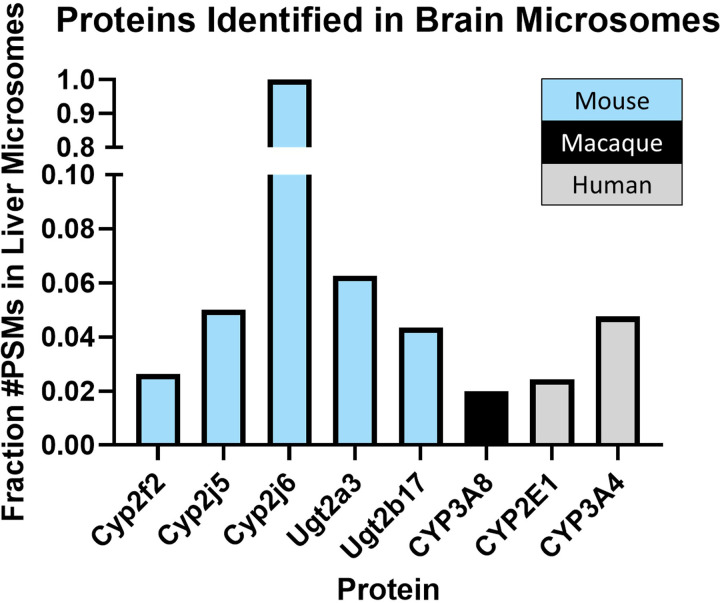
Proteins detected in the brain microsomes exhibited a fraction of the peptide spectral matches identified for the same proteins in the liver microsomes. Untargeted proteomics comparing liver vs. brain microsomes from mouse, cynomolgus macaque, and human revealed the presence of several drug metabolizing enzymes, each with a single peptide spectral match. These matches were only a fraction of the peptide spectral matches observed in the liver microsomes for all proteins except for Cyp2j6 in the mouse microsomes.

A global untargeted proteomics experiment was first used to determine the expression of P450s and UGTs in liver microsomes versus brain microsomes isolated from mouse, cynomolgus macaque, or human tissue. Relative comparisons were achieved by using the number of peptide spectral matches identified for each protein, a label-free, semiquantitative way to measure protein abundance (Lundgren et al., 2010). In the mouse brain microsomes, a single unique peptide spectral match was found for Cyp2f2, Cyp2j5, Cyp2j6, Ugt2a3, and Ugt2b17. For all but Cyp2j6, these matches were only a fraction of those observed in liver microsomes (Fig. 6). Due to high sequence homology between these enzymes, additional nonunique peptide spectral matches were found for Cyp2d10, Cyp2d11, and Cyp2d26, where a single peptide corresponding to all three proteins was observed. UGTs 1a1, 1a2, 1a6, and 1a9 also shared a single peptide spectral match. Conversely, multiple peptides corresponding to each of these proteins were revealed in the mouse liver microsomes. In the microsomes from cynomolgus macaque, one unique peptide matching CYP3A8 was identified in the brain microsomes compared with 58 peptide spectral matches for CYP3A8 in the liver microsomes. In the human brain microsomes, CYP2E1 and CYP3A4 each showed a single unique peptide spectral match compared with 41 and 21 unique peptide matches in the liver microsomes, respectively (Fig. 6). A nonunique peptide corresponding to UGT1A1, UGT1A3, UGT1A4, UGT1A5, UGT1A6, UGT1A7, UGT1A8, UGT1A9, or UGT1A10 was also noted in the human brain microsomes.

### Targeted Proteomics Identified Several P450s and UGTs in Brain Microsomes

**Fig. 7.**
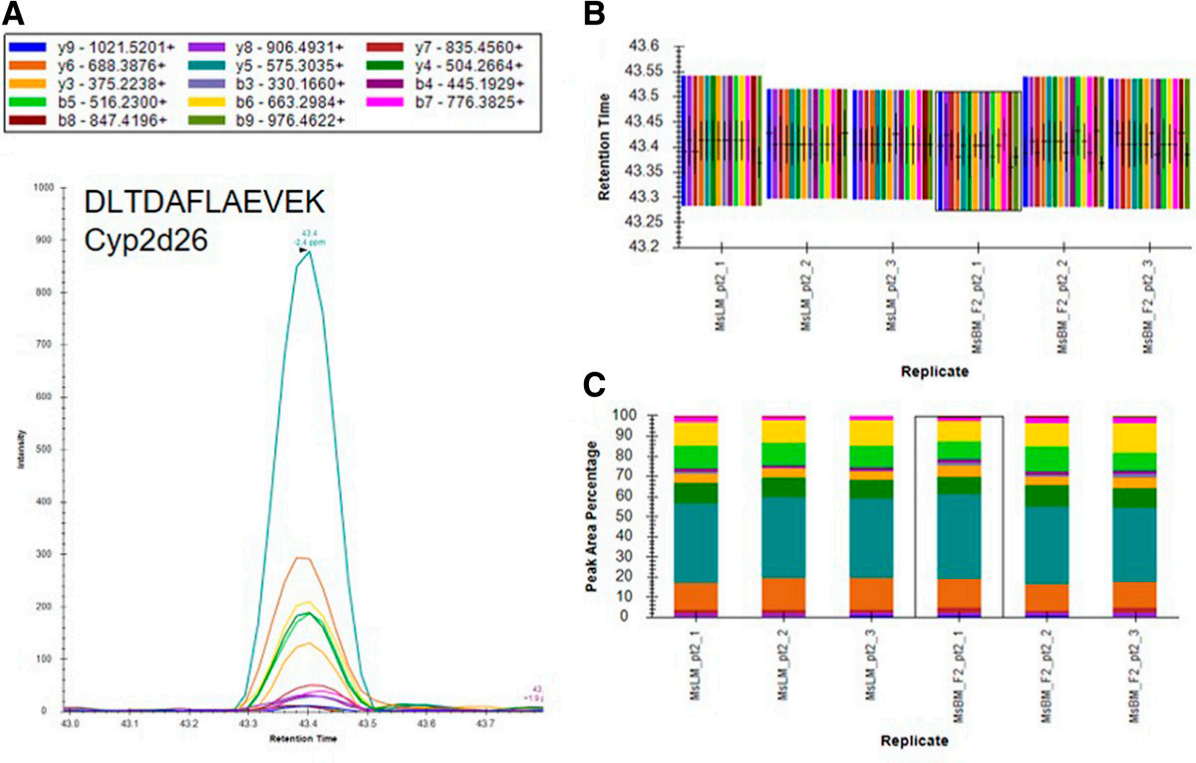
Identification of Cyp2d26 in mouse brain microsomes using a targeted proteomics approach. Mouse brain microsomes were digested and resulting peptides were fractionated before using targeted proteomics to show the presence of Cyp2d26 (A). Samples were run in triplicate and peptide identifications were validated by comparing retention time (B) and peak area percentage of fragment ions (C) between mouse brain microsomes (MsBM) and mouse liver microsomes (MsLM). Peptide identifications for Cyp1a1, Cyp2c39, Cyp2d10, Cy2d11, Cyp4a10, and Cyp7b1 are illustrated in Supplemental Fig. 1.

**Fig. 8.**
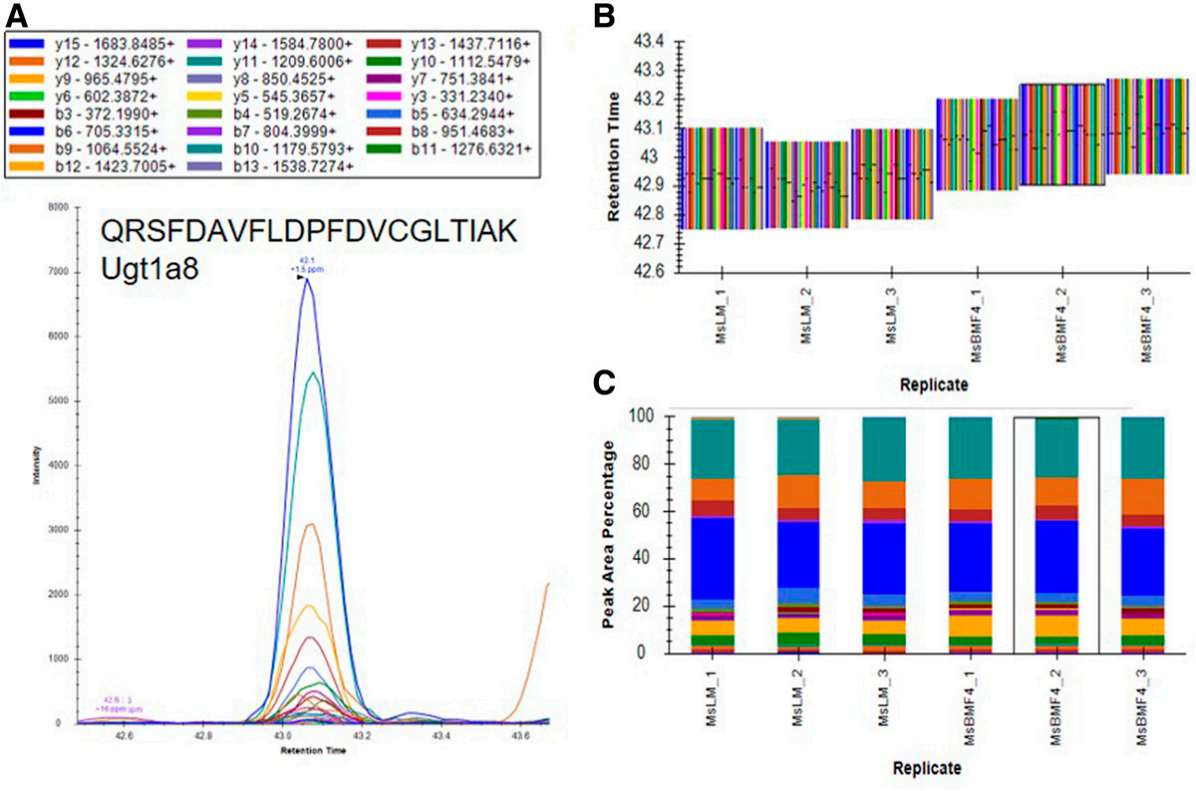
Identification of Ugt1a8 in mouse brain microsomes using a targeted proteomics approach. Mouse brain microsomes were digested and resulting peptides were fractionated before using targeted proteomics to show the presence of Ugt1a8 (A). Samples were run in triplicate and peptide identifications were validated by comparing retention time (B) and peak area percentage of fragment ions (C) between mouse brain microsomes (MsBM) and mouse liver microsomes (MsLM). Peptide identifications for Ugt1a1, Ugt1a2, Ugt1a5, Ugt1a7, Ugt1a9, Ugt1a10, Ugt2a1, Ugt2a2, Ugt2a3, Ugt2b1, Ugt2b17, Ugt2b34, Ugt2b35, and Ugt3a1 are illustrated in Supplemental Fig. 2.

**Fig. 9.**
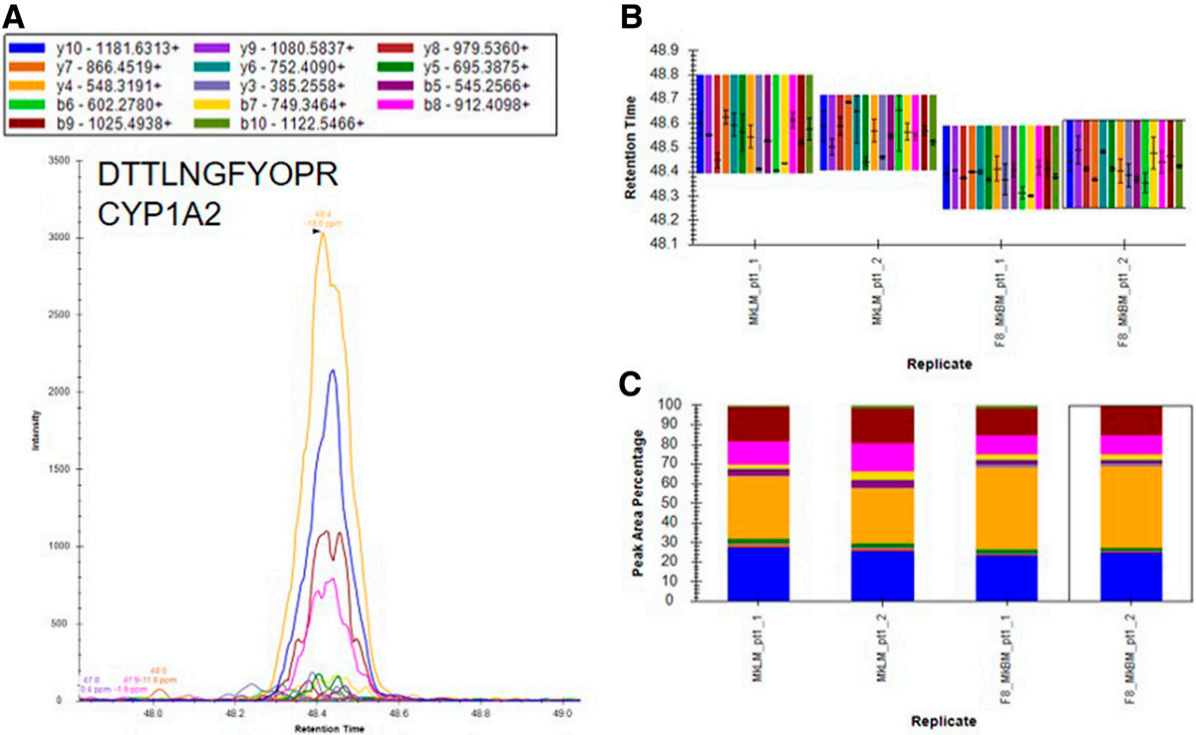
Identification of CYP1A2 in cynomolgus macaque brain microsomes using a targeted proteomics approach. Cynomolgus macaque brain microsomes were digested and resulting peptides were fractionated before using targeted proteomics to show the presence of CYP1A2 (A). Samples were run in duplicate and peptide identifications were validated by comparing retention time (B) and peak area percentage of fragment ions (C) between macaque brain microsomes (MkBM) and macaque liver microsomes (MkLM). Peptide identifications for CYP1A1, CYP1B1, CYP2E1, CYP2F1, CYP2R1, CYP2U1, CYP2W1, CYP4F12, CYP4F22, CYP11B2, CYP20A1, CYP21A2, CYP27A1, and CYP27C1 are illustrated in Supplemental Fig. 3.

**Fig. 10.**
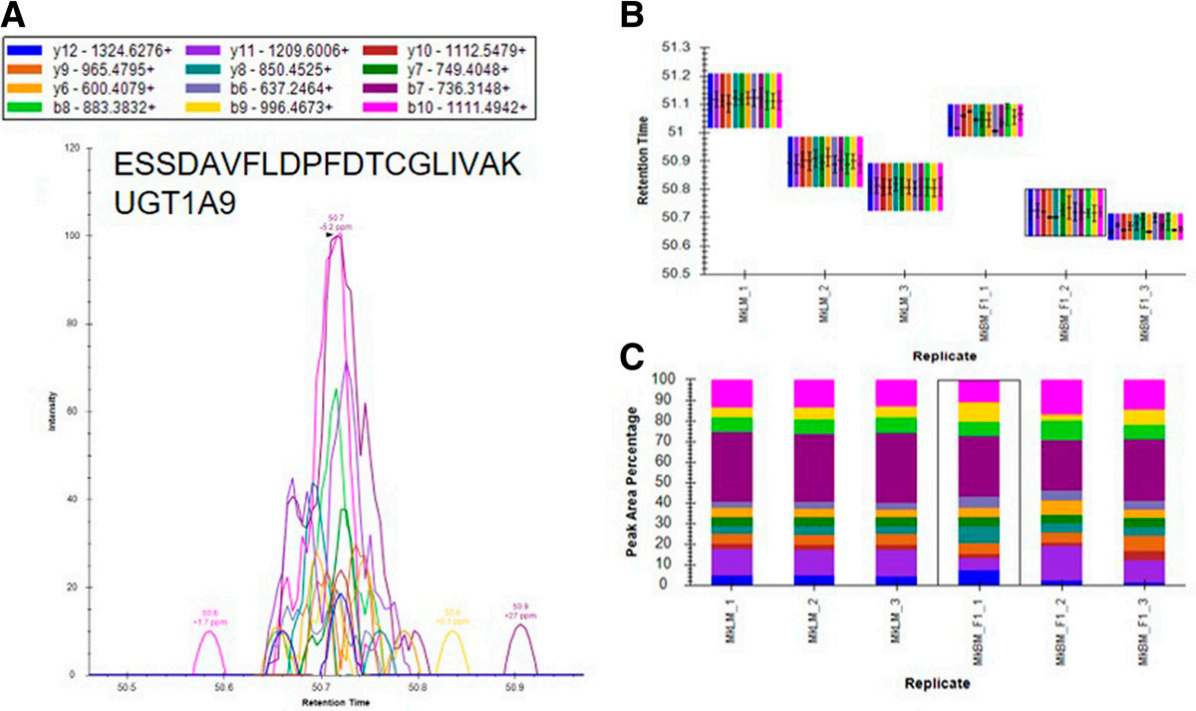
Identification of UGT1A9 in cynomolgus macaque brain microsomes using a targeted proteomics approach. Cynomolgus macaque brain microsomes were digested and resulting peptides were fractionated before using targeted proteomics to show the presence of UGT1A9 (A). Samples were run in triplicate and peptide identifications were validated by comparing retention time (B) and peak area percentage of fragment ions (C) between macaque brain microsomes (MkBM) and macaque liver microsomes (MkLM). Peptide identifications for UGT1A10, UGT2B9, and UGT2B20 are illustrated in Supplemental Fig. 4.

**Fig. 11.**
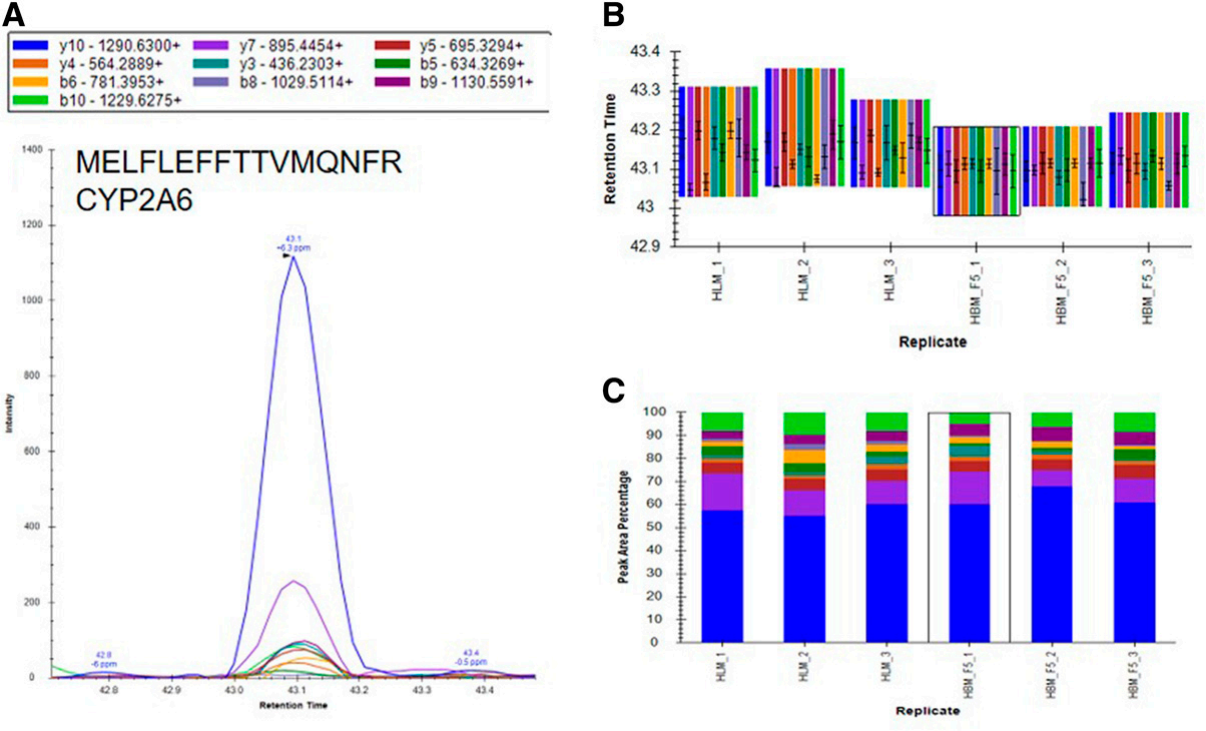
Identification of CYP2A6 inhuman brain microsomes using a targeted proteomics approach. Human brain microsomes were digested and resulting peptides were fractionated before using targeted proteomics to show the presence of CYP2A6 (A). Samples were run in duplicate or triplicate and peptide identifications were validated by comparing retention time (B) and peak area percentage of fragment ions (C) between human brain microsomes (HBM) and human liver microsomes (HLM). Peptide identifications for CYP1A2, CYP2B6, CYP2D6, CYP2E1, CYP2J2, CYP3A4, CYP4A11, CYP4F3, CYP4F12, and CYP20A1 are illustrated in Supplemental Fig. 5.

**Fig. 12.**
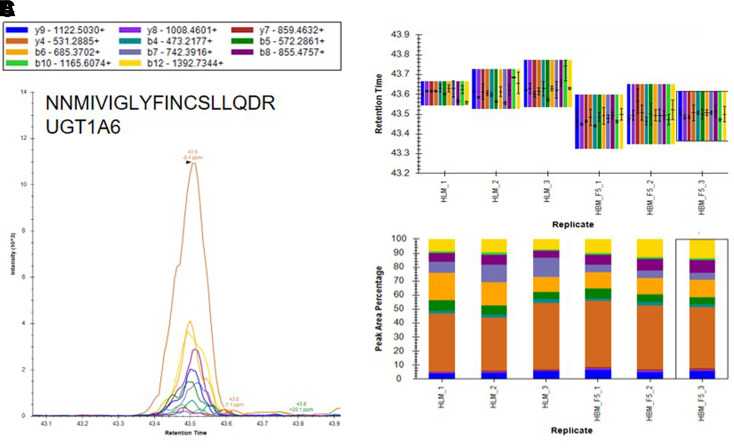
Identification of UGT1A6 in human brain microsomes using a targeted proteomics approach. Human brain microsomes were digested and resulting peptides were fractionated before using a targeted proteomics approach to show the presence of UGT1A6 (A). Samples were run in duplicate or triplicate and peptide identifications were validated by comparing retention time (B) and peak area percentage of fragment ions (C) between human brain microsomes (HBM) and human liver microsomes (HLM). Peptide identifications for UGT1A1, UGT1A4, UGT1A5, UGT1A6, UGT1A8, UGT2A1, UGT2A2, UGT2B4, UGT2B7, UGT2B17, and UGT2B28 are illustrated in Supplemental Fig. 6.

Based on the results of our microsomal assays indicating the presence of UGTs in the macaque and human brain microsomes and the lack of identification of these enzymes in our untargeted proteomics results, we sought a method to identify the low abundance proteins of interest in the brain microsome samples. It is well established that targeted proteomic methods have a lower limit of detection than global analysis techniques (Domon and Aebersold, 2010). A recent advance in targeted mass spectrometry allows the simultaneous generation of multiple fragment ions from a peptide that can be identified with high resolution and high mass accuracy. To develop targeted proteomics methods, we created a target list of unique peptides to be used in the identification of P450s and UGTs in mouse, cynomolgus macaque, and human brain microsomes. To be considered an identification, each unique peptide had a mass error ≤5 ppm, a minimum of three fragment ions with intensities at least three times above the noise, and an acceptable peak shape. Only proteins from which quality peptides could be reliably detected in the liver microsomes were targeted in these experiments. Using the target lists, scheduled ionization, and these criteria, we identified peptides from several different P450s and UGTs (Table 1). In the mouse brain microsomes, 10 peptides corresponding to seven different P450s—1a1, 2c39, 2d10, 2d11, 2d26, 4a10, and 7b1 (Fig. 7; Supplemental Fig. 1; Supplemental Table 7)—and 25 peptides matching 15 UGTs—1a1, 1a2, 1a5, 1a7, 1a8, 1a9, 1a10, 2a1, 2a2, 2a3, 2b17, 2b34, 2b35, and 3a1 (Fig. 8; Supplemental Fig. 2; Supplemental Table 7)—were observed. In the cynomolgus macaque brain microsomes, 18 unique peptides corresponding to 15 P450s—1A1, 1A2, 1B1, 2C18, 2E1, 2F1, 2F12, 2R1, 2U1, 2W1, 11B2, 20A1, 21A2, 27A1, and 27C1 (Fig. 9; Supplemental Fig. 3; Supplemental Table 8)—and four peptides matching four UGTs—1A9, 1A10, 2B9, and 2B20—were detected (Fig. 10; Supplemental Fig. 4; Supplemental Table 8). In the human brain microsomes, 15 peptides matching 11 different P450s—1A2, 2A6, 2B6, 2D6, 2E1, 2J2, 3A4, 4A11, 4F3, 4F12, and 20A1 (Fig. 11; Supplemental Fig. 5; Supplemental Table 9)—and 14 peptides matching 11 different UGTs—1A1, 1A4, 1A5, 1A6, 1A8, 2A1, 2A2, 2B4, 2B7, 2B17, and 2B28—were observed (Fig. 12; Supplemental Fig. 6; Supplemental Table 9).

**TABLE 1:** Summary of P450s and UGTs detected in brain microsomes

Mouse P450s	1a1, 2c39, 2d10, 2d11, 2d26, 4a10, 7b1
Macaque P450s	1A1, 1A2, 1B1, 2E1, 2F1, 2F12, 2R1, 2U1, 2W1, 4F12, 11B2 20A1, 21A2, 27A1, 27C1
Human P450s	1A2, 2A6, 2B6, 2C9, 2E1, 2J2, 3A4, 4A11, 4F3, 4F11, 4F12, 20A1
Mouse UGTs	1a1, 1a2, 1a5, 1a7, 1a8, 1a9, 1a10, 2a1, 2a2, 2a3, 2b17, 2b34, 2b35, 3a1
Macaque UGTs	1A9, 1A10, 2B9, 2B20
Human UGTs	1A1, 1A4, 1A5, 1A6, 1A8, 2A1, 2A2, 2B4, 2B7, 2B17, 2B28

## Discussion

This work aimed to deepen the understanding of drug metabolism in the brain, specifically for EFV. Our in vitro studies indicate that the brain can metabolize EFV and glucuronidate its P450-dependent metabolites. Moreover, we identified species differences in the direct glucuronidation of EFV, where brain microsomes from cynomolgus macaques formed EFV-G but the other brain microsomes did not. We also uncovered cell type–specific metabolism of EFV in mouse neural cells. Microglia metabolized EFV to 8-OHEFV, whereas cortical neurons and astrocytes glucuronidated both 8-OHEFV and 8,14-diOHEFV. This evidence indicates that local biotransformation of EFV and its P450-dependent metabolites can occur in brain and that this metabolism can vary by cell type and species. EFV metabolism in the brain could have consequences in both the sphere of drug efficacy and neurotoxicity.

Clinically, EFV is associated with a range of neurologic adverse events, with symptoms such as dizziness, depression, impaired concentration, disordered sleep, and anxiety occurring in up to 70% of patients (Fumaz et al., 2002; Gutiérrez et al., 2005; Checa et al., 2020). Although these symptoms often subside over time, central nervous system (CNS) adverse events lead to regimen interruption and lower quality of life (Fumaz et al., 2002; Gutiérrez et al., 2005; Hawkins et al., 2005; Vera et al., 2019). Both EFV and 8-OHEFV reach the CSF at approximately 10 ng/ml and 3 ng/ml, respectively. (Tashima et al., 1999; Best et al., 2011; Avery et al., 2013a,b). Though EFV is 99.5% protein bound, similar unbound drug concentrations between plasma and CSF suggests unbound efavirenz can passively enter the CNS (Best et al., 2011; Avery et al., 2013a). Although EFV has been noted to induce P-glycoprotein, it is not a substrate of the transporter (Dirson et al., 2006; Chan et al., 2013). EFV is found in the CSF at concentrations above the IC_95_ for wild-type HIV-1 (Tashima et al., 1999; Best et al., 2011; Calcagno et al., 2015), contradicting the notion that the ongoing prevalence of HAND is a result of poor antiretroviral penetration into the CNS. Additionally, the 8-OHEFV concentrations found in human CSF are similar to the dose that induced in vitro toxicity in rat hippocampal neurons (Tovar-y-Romo et al., 2012; Avery et al., 2013b). However, phase 2 metabolites, 8-OHEFV-G and 8-OHEFV-sulfate, were noted at even higher concentrations: 15–56 ng/ml and 0–29 ng/ml, respectively (Aouri et al., 2016; Nightingale et al., 2016). Nightingale et al. (2016) note that these higher concentrations could be a result of local metabolism, as the percentage of free EFV is not significantly greater in the CSF than it is in the plasma. The slow metabolizing CYP2B6 (G516T) T/T genotype is associated with higher plasma and CSF concentrations of EFV (Haas et al., 2004; Nightingale et al., 2016) as well as late onset efavirenz neurotoxicity syndrome (van Rensburg et al., 2022). Further, in silico modeling indicates that measurements of EFV in the CSF likely underrepresent the EFV penetration into the brain (Curley et al., 2016). These predictions were confirmed in a study that analyzed the postmortem brain tissue of patients taking EFV and noted an average tissue concentration of 35.9 ng/ml EFV compared with 15.9 ng/ml in the CSF (Aouri et al., 2016; Ferrara et al., 2020). Local brain metabolism of EFV, particularly glucuronidation, could be advantageous in terms of neurotoxicity but detrimental to efficacy at the site of infection. Several studies have examined drug-metabolizing enzymes in the brain, but few have measured these drug-metabolizing enzymes at the protein level. Using a proteomics approach, we sought to identify the P450s and UGTs present in brain microsomes.

The mRNA transcripts of several P450s have been reported in the human brain, with 1B1, 2D6, 2E1, 2J2, and 46A1 being the most abundant (Dutheil et al., 2009). CYP2D6 metabolizes endogenous substances like dopamine and serotonin as well as a number of different drugs that target the CNS such as opioids, neuroleptics, antidepressants, selective serotonin reuptake inhibitors, and antiemetics (Hiroi et al., 1998; Yu et al., 2003; Wang et al., 2009; Bromek et al., 2011; Haduch et al., 2015). Furthermore, lower levels of CYP2D6 in the brain have been associated with Parkinson’s disease (Mann et al., 2012). CYP2E1 is known to both metabolize and be induced by ethanol in the brain (Zimatkin et al., 2006; Zhong et al., 2012; Ferguson et al., 2013). In the brain, CYP2B6 has been implicated in nicotine metabolism (Garcia et al., 2015), and nicotine has been shown to induce CYP2B6 expression (Miksys et al., 2003; Lee et al., 2006; Ferguson et al., 2013). CYP2B6 is also known to metabolize the antidepressant and smoking cessation aid bupropion (Hesse et al., 2000), which has been observed, along with its metabolites, in brain tissue (Suckow et al., 1986). CYP46A1 catalyzes cholesterol 24-hydroxylation and is important in regulating cholesterol homeostasis in the brain, whereas dysregulation has been linked to neurodegeneration (Djelti et al., 2015). EFV is known to be a CYP46A1 activator at low doses and has been explored for the treatment of Alzheimer’s disease (Petrov et al., 2019; Mast et al., 2020). Lastly, human brain microsomes have also been noted to exhibit P450 activity by CYP3A4, CYP2D6, or CYP2C19 through the demethylation of the antidepressant amitriptyline (Voirol et al., 2000).

Using targeted P450 proteomics, we identified a number of P450s in mouse, cynomolgus macaque, and human brain microsomes. Several studies have shown the presence of P450s, either at the mRNA or protein level, in murine brain (Stapleton et al., 1995; Choudhary et al., 2005; Renaud et al., 2011; Hersman and Bumpus, 2014; Stamou et al., 2014; Yamaori et al., 2017). Our identification of Cyp1a1, Cyp2c39, Cyp2d10, Cyp2d26, Cyp4a10, and Cyp7b1 coincides with these studies (Supplemental Table 10). Detection of CYP1A1 and CYP2E1 in macaque brain microsomes was commensurate with a previous mRNA-based study (Uno and Yamazaki, 2020b), whereas the remaining 13 P450s that we identified in macaque brain microsomes (P450s 1A2, 1B1, 2C18, 2F1, 2F12, 2R1, 2U1, 11B2, 20A1, 21A2, 27A1, and 27C1) have not been previously noted in the brain (Supplemental Table 11). P450s in the human brain have also been previously reported (McFadyen et al., 1998; Yun et al., 1998; Gervot et al., 1999; Upadhya et al., 2000; Miksys et al., 2002; Stark et al., 2008; Booth Depaz et al., 2015), including CYP1A2, CYP2B6, CYP2C9 CYP2E1, CYP3A4, and CYP20A1, which were identified in the present study using targeted proteomics (Supplemental Table 12). We additionally identified peptides corresponding to CYP2A6, CYP2J2, CYP4A11, CYP4F3, and CYP4F12.

Less is known about UGTs in the brain compared with P450s. However, UGTs have been shown to be involved in the metabolism of both endogenous substrates, such as dopamine and serotonin, and xenobiotics, such as morphine, in the brain (Wahlström et al., 1988; Uutela et al., 2009; Ouzzine et al., 2014). We identified 14 different UGTs in the mouse brain microsomes, 12 of which have been detected at the mRNA level in previous studies (Buckley and Klaassen, 2007; Heydel et al., 2010; Uchihashi et al., 2013), whereas the other two UGTs (Ugt1a9 and Ugt2b17) have not been previously reported in murine brain (Supplemental Table 10). The mRNA expression of UGTs 1A1, 1A9, 1A10, 2B9, 2B18, 2B19, 2B23, 3A2, and 8A1 has been reported in cynomolgus macaque brain tissue (Uno and Yamazaki, 2020a,b), of which we observed UGT1A9, UGT1A10, and UGT2B9 protein in macaque brain microsomes (Supplemental Table 11). We also identified UGT2B20, and to our knowledge, UGT protein and/or activity have yet to be reported in cynomolgus macaque brain. Lastly, the mRNA transcript of UGTs 1A1, 1A3, 1A4, 1A5, 1A6, 1A7, 1A10, 2A1, 2B7, and 2B17 have been previously identified in human brain tissue (Jedlitschky et al., 1999; King et al., 1999; Ohno and Nakajin, 2009; Court et al., 2012) and UGT1A4 protein has been reported as well (Ghosh et al., 2010), corresponding with our study (Supplemental Table 12). Using the targeted proteomics method that we developed, UGTs 1A8, 2A1, 2A2, 2B4, and 2B28 were also identified. These results are consistent with our microsomal metabolism data, as UGT1A1 and UGT1A8 have each been reported to carry out the glucuronidation of the P450-dependent metabolites of EFV (Bae et al., 2011).

In summary, the in vitro metabolism of EFV observed in this study and the identification of P450 and UGT protein in the brain microsomes of mice, cynomolgus macaques, and humans lend novel insight into the local metabolism of the EFV, which would have implications for combatting HIV in the brain. Biotransformation of EFV in the brain would reduce active drug concentration while potentially modulating neurotoxicity. The presence of drug-metabolizing enzymes in the brain at the protein level has not been previously well established, but the targeted methods used can be applied to probing low-abundance P450s and UGTs in other tissues, contributing to research regarding tissue-specific pharmaceuticals or toxicity. The proteomic data presented in this study represent a fundamental asset in understanding and predicting P450 and UGT metabolism of any drug that crosses the blood-brain barrier and can guide future activity-based studies.

## Abbreviations

cART: combination antiretroviral therapy
CNS: central nervous system
CSF: cerebrospinal fluid
8, 14-diOHEFV: 8, 14-dihydroxyefavirenz
8, 14-diOHEFV-G: 8, 14-dihydroxyefavirenz glucuronide
EFV: efavirenz
EFV-G: efavirenz glucuronide
HAND: HIV-associated neurocognitive disorder
HIV: human immunodeficiency virus
MS: mass spectrometry
m/z: mass-to-charge ratio
8-OHEFV: 8-hydroxyefavirenz
8-OHEFV-G: 8-hydroxyefavirenz glucuronide
P450: cytochrome P450
UDPGA: UDP glucuronic acid
UGT: UDP-glucuronosyltransferase
uHPLC-HRMS: ultrahigh performance liquid chromatography high-resolution mass spectrometry

## References

[B1] Akiyama H Jalloh S Park S Lei M Mostoslavsky G Gummuluru S (2020) Expression of HIV-1 intron-containing RNA in microglia induces inflammatory responses. J Virol 95:e01385–20. 10.1128/JVI.01386-20 PMC8092841 33298546

[B2] Aljawai Y Richards MH Seaton MS Narasipura SD Al-Harthi L (2014) β-Catenin/TCF-4 signaling regulates susceptibility of macrophages and resistance of monocytes to HIV-1 productive infection. Curr HIV Res 12:164–173. 24862328 10.2174/1570162x12666140526122249 PMC4331035

[B3] Antinori A Arendt G Becker JT Brew BJ Byrd DA Cherner M Clifford DB Cinque P Epstein LG Goodkin K, (2007) Updated research nosology for HIV-associated neurocognitive disorders. Neurology 69:1789–1799. 17914061 10.1212/01.WNL.0000287431.88658.8b PMC4472366

[B4] Aouri M Barcelo C Ternon B Cavassini M Anagnostopoulos A Yerly S Hugues H Vernazza P Günthard HF Buclin T, ; Swiss HIV Cohort Study (2016) In vivo profiling and distribution of known and novel phase I and phase II metabolites of efavirenz in plasma, urine, and cerebrospinal fluid. Drug Metab Dispos 44:151–161. 26553012 10.1124/dmd.115.065839

[B5] Avery LB Sacktor N McArthur JC Hendrix CW (2013a) Protein-free efavirenz concentrations in cerebrospinal fluid and blood plasma are equivalent: applying the law of mass action to predict protein-free drug concentration. Antimicrob Agents Chemother 57:1409–1414. 23295919 10.1128/AAC.02329-12 PMC3591913

[B6] Avery LB VanAusdall JL Hendrix CW Bumpus NN (2013b) Compartmentalization and antiviral effect of efavirenz metabolites in blood plasma, seminal plasma, and cerebrospinal fluid. Drug Metab Dispos 41:422–429. 23166317 10.1124/dmd.112.049601 PMC3558859

[B7] Bae SK Jeong YJ Lee C Liu KH (2011) Identification of human UGT isoforms responsible for glucuronidation of efavirenz and its three hydroxy metabolites. Xenobiotica 41:437–444. 21319958 10.3109/00498254.2011.551849

[B8] Best BM Koopmans PP Letendre SL Capparelli EV Rossi SS Clifford DB Collier AC Gelman BB Mbeo G McCutchan JA, ; CHARTER Group (2011) Efavirenz concentrations in CSF exceed IC50 for wild-type HIV. J Antimicrob Chemother 66:354–357. 21098541 10.1093/jac/dkq434 PMC3019085

[B9] Booth Depaz IM Toselli F Wilce PA Gillam EM (2015) Differential expression of cytochrome P450 enzymes from the CYP2C subfamily in the human brain. Drug Metab Dispos 43:353–357. 25504503 10.1124/dmd.114.061242 PMC6067382

[B10] Bromek E Haduch A Gołembiowska K Daniel WA (2011) Cytochrome P450 mediates dopamine formation in the brain in vivo. J Neurochem 118:806–815. 21651557 10.1111/j.1471-4159.2011.07339.x

[B11] Buckley DB Klaassen CD (2007) Tissue- and gender-specific mRNA expression of UDP-glucuronosyltransferases (UGTs) in mice. Drug Metab Dispos 35:121–127. 17050650 10.1124/dmd.106.012070

[B12] Calcagno A Simiele M Alberione MC Bracchi M Marinaro L Ecclesia S Di Perri G D’Avolio A Bonora S (2015) Cerebrospinal fluid inhibitory quotients of antiretroviral drugs in HIV-infected patients are associated with compartmental viral control. Clin Infect Dis 60:311–317. 25281609 10.1093/cid/ciu773

[B13] Cenker JJ Stultz RD McDonald D (2017) Brain microglial cells are highly susceptible to HIV-1 infection and spread. AIDS Res Hum Retroviruses 33:1155–1165. 28486838 10.1089/aid.2017.0004 PMC5665495

[B14] Chaganti J Marripudi K Staub LP Rae CD Gates TM Moffat KJ Brew BJ (2019) Imaging correlates of the blood-brain barrier disruption in HIV-associated neurocognitive disorder and therapeutic implications. AIDS 33:1843–1852. 31274535 10.1097/QAD.0000000000002300

[B15] Chan GN Patel R Cummins CL Bendayan R (2013) Induction of P-glycoprotein by antiretroviral drugs in human brain microvessel endothelial cells. Antimicrob Agents Chemother 57:4481–4488. 23836171 10.1128/AAC.00486-13 PMC3754350

[B16] Checa A Castillo A Camacho M Tapia W Hernandez I Teran E (2020) Depression is associated with efavirenz-containing treatments in newly antiretroviral therapy initiated HIV patients in Ecuador. AIDS Res Ther 17:47. 32727488 10.1186/s12981-020-00303-1 PMC7391584

[B17] Chivero ET Guo ML Periyasamy P Liao K Callen SE Buch S (2017) HIV-1 Tat primes and activates microglial NLRP3 inflammasome-mediated neuroinflammation. J Neurosci 37:3599–3609. 28270571 10.1523/JNEUROSCI.3045-16.2017 PMC5373137

[B18] Choudhary D Jansson I Stoilov I Sarfarazi M Schenkman JB (2005) Expression patterns of mouse and human CYP orthologs (families 1-4) during development and in different adult tissues. Arch Biochem Biophys 436:50–61. 15752708 10.1016/j.abb.2005.02.001

[B19] Ciavatta VT Bichler EK Speigel IA Elder CC Teng SL Tyor WR García PS (2017) In vitro and ex vivo neurotoxic effects of efavirenz are greater than those of other common antiretrovirals. Neurochem Res 42:3220–3232. 28770436 10.1007/s11064-017-2358-x

[B20] Court MH Zhang X Ding X Yee KK Hesse LM Finel M (2012) Quantitative distribution of mRNAs encoding the 19 human UDP-glucuronosyltransferase enzymes in 26 adult and 3 fetal tissues. Xenobiotica 42:266–277. 21995321 10.3109/00498254.2011.618954

[B21] Curley P Rajoli RK Moss DM Liptrott NJ Letendre S Owen A Siccardi M (2016) Efavirenz is predicted to accumulate in brain tissue: an in silico, in vitro, and in vivo investigation. Antimicrob Agents Chemother 61:e01841–16. 27799216 10.1128/AAC.01841-16 PMC5192136

[B22] Dirson G Fernandez C Hindlet P Roux F German-Fattal M Gimenez F Farinotti R (2006) Efavirenz does not interact with the ABCB1 transporter at the blood-brain barrier. Pharm Res 23:1525–1532. 16779703 10.1007/s11095-006-0279-5

[B23] Djelti F Braudeau J Hudry E Dhenain M Varin J Bièche I Marquer C Chali F Ayciriex S Auzeil N, (2015) CYP46A1 inhibition, brain cholesterol accumulation and neurodegeneration pave the way for Alzheimer’s disease. Brain 138:2383–2398. 26141492 10.1093/brain/awv166

[B24] Domon B Aebersold R (2010) Options and considerations when selecting a quantitative proteomics strategy. Nat Biotechnol 28:710–721. 20622845 10.1038/nbt.1661

[B25] Dutheil F Dauchy S Diry M Sazdovitch V Cloarec O Mellottée L Bièche I Ingelman-Sundberg M Flinois JP de Waziers I, (2009) Xenobiotic-metabolizing enzymes and transporters in the normal human brain: regional and cellular mapping as a basis for putative roles in cerebral function. Drug Metab Dispos 37:1528–1538. 19359404 10.1124/dmd.109.027011

[B26] Eugenin EA King JE Nath A Calderon TM Zukin RS Bennett MV Berman JW (2007) HIV-tat induces formation of an LRP-PSD-95- NMDAR-nNOS complex that promotes apoptosis in neurons and astrocytes. Proc Natl Acad Sci USA 104:3438–3443. 17360663 10.1073/pnas.0611699104 PMC1805607

[B27] Ferguson CS Miksys S Palmour RM Tyndale RF (2013) Ethanol self-administration and nicotine treatment induce brain levels of CYP2B6 and CYP2E1 in African green monkeys. Neuropharmacology 72:74–81. 23639433 10.1016/j.neuropharm.2013.04.022

[B28] Ferrara M Bumpus NN Ma Q Ellis RJ Soontornniyomkij V Fields JA Bharti A Achim CL Moore DJ Letendre SL (2020) Antiretroviral drug concentrations in brain tissue of adult decedents. AIDS 34:1907–1914. 32694413 10.1097/QAD.0000000000002628 PMC10768889

[B29] Fumaz CR Tuldrà A Ferrer MJ Paredes R Bonjoch A Jou T Negredo E Romeu J Sirera G Tural C, (2002) Quality of life, emotional status, and adherence of HIV-1-infected patients treated with efavirenz versus protease inhibitor-containing regimens. J Acquir Immune Defic Syndr 29:244–253. 11873073 10.1097/00042560-200203010-00004

[B30] Funes HA Blas-Garcia A Esplugues JV Apostolova N (2015) Efavirenz alters mitochondrial respiratory function in cultured neuron and glial cell lines. J Antimicrob Chemother 70:2249–2254. 25925594 10.1093/jac/dkv098

[B31] Gandhi N Saiyed ZM Napuri J Samikkannu T Reddy PV Agudelo M Khatavkar P Saxena SK Nair MP (2010) Interactive role of human immunodeficiency virus type 1 (HIV-1) clade-specific Tat protein and cocaine in blood-brain barrier dysfunction: implications for HIV-1-associated neurocognitive disorder. J Neurovirol 16:294–305. 20624003 10.3109/13550284.2010.499891

[B32] Garcia KL Coen K Miksys S Lê AD Tyndale RF (2015) Effect of brain CYP2B inhibition on brain nicotine levels and nicotine self-administration. Neuropsychopharmacology 40:1910–1918. 25652250 10.1038/npp.2015.40 PMC4839514

[B33] Gervot L Rochat B Gautier JC Bohnenstengel F Kroemer H de Berardinis V Martin H Beaune P de Waziers I (1999) Human CYP2B6: expression, inducibility and catalytic activities. Pharmacogenetics 9:295–306. 10471061

[B34] Ghosh C Gonzalez-Martinez J Hossain M Cucullo L Fazio V Janigro D Marchi N (2010) Pattern of P450 expression at the human blood-brain barrier: roles of epileptic condition and laminar flow. Epilepsia 51:1408–1417. 20074231 10.1111/j.1528-1167.2009.02428.x PMC3386640

[B35] Grilo NM Correia MJ Sequeira C Harjivan SG Caixas U Diogo LN Marques MM Monteiro EC Antunes AM Pereira SA (2016) Efavirenz biotransformation as an up-stream event of mood changes in HIV-infected patients. Toxicol Lett 260:28–35. 27543169 10.1016/j.toxlet.2016.08.009

[B36] Gutiérrez F Navarro A Padilla S Antón R Masiá M Borrás J Martín-Hidalgo A (2005) Prediction of neuropsychiatric adverse events associated with long-term efavirenz therapy, using plasma drug level monitoring. Clin Infect Dis 41:1648–1653. 16267739 10.1086/497835

[B37] Haas DW Ribaudo HJ Kim RB Tierney C Wilkinson GR Gulick RM Clifford DB Hulgan T Marzolini C Acosta EP (2004) Pharmacogenetics of efavirenz and central nervous system side effects: an Adult AIDS Clinical Trials Group study. AIDS 18:2391–2400. 15622315

[B38] Haduch A Bromek E Kot M Kamińska K Gołembiowska K Daniel WA (2015) The cytochrome P450 2D-mediated formation of serotonin from 5-methoxytryptamine in the brain in vivo: a microdialysis study. J Neurochem 133:83–92. 25581337 10.1111/jnc.13031

[B39] Hakkers CS Hermans AM van Maarseveen EM Teunissen CE Verberk IMW Arends JE Hoepelman AIM (2020) High efavirenz levels but not neurofilament light plasma levels are associated with poor neurocognitive functioning in asymptomatic HIV patients. J Neurovirol 26:572–580. 32524424 10.1007/s13365-020-00860-1 PMC7438296

[B40] Hawkins T Geist C Young B Giblin A Mercier RC Thornton K Haubrich R (2005) Comparison of neuropsychiatric side effects in an observational cohort of efavirenz- and protease inhibitor-treated patients. HIV Clin Trials 6:187–196. 16214735 10.1310/92vr-fp24-j8ga-b49q

[B41] Hersman EM Bumpus NN (2014) A targeted proteomics approach for profiling murine cytochrome P450 expression. J Pharmacol Exp Ther 349:221–228. 24594750 10.1124/jpet.113.212456 PMC3989799

[B42] Hesse LM Venkatakrishnan K Court MH von Moltke LL Duan SX Shader RI Greenblatt DJ (2000) CYP2B6 mediates the in vitro hydroxylation of bupropion: potential drug interactions with other antidepressants. Drug Metab Dispos 28:1176–1183. 10997936

[B43] Heydel JM Holsztynska EJ Legendre A Thiebaud N Artur Y Le Bon AM (2010) UDP-glucuronosyltransferases (UGTs) in neuro-olfactory tissues: expression, regulation, and function. Drug Metab Rev 42:74–97. 20067364 10.3109/03602530903208363

[B44] Hiroi T Imaoka S Funae Y (1998) Dopamine formation from tyramine by CYP2D6. Biochem Biophys Res Commun 249:838–843. 9731223 10.1006/bbrc.1998.9232

[B45] Jedlitschky G Cassidy AJ Sales M Pratt N Burchell B (1999) Cloning and characterization of a novel human olfactory UDP-glucuronosyltransferase. Biochem J 340:837–843. 10359671 PMC1220318

[B46] Ji HY Lee H Lim SR Kim JH Lee HS (2012) Effect of efavirenz on UDP-glucuronosyltransferase 1A1, 1A4, 1A6, and 1A9 activities in human liver microsomes. Molecules 17:851–860. 22252501 10.3390/molecules17010851 PMC6268312

[B47] King CD Rios GR Assouline JA Tephly TR (1999) Expression of UDP-glucuronosyltransferases (UGTs) 2B7 and 1A6 in the human brain and identification of 5-hydroxytryptamine as a substrate. Arch Biochem Biophys 365:156–162. 10222050 10.1006/abbi.1999.1155

[B48] Ko A Kang G Hattler JB Galadima HI Zhang J Li Q Kim W-K (2019) Macrophages but not astrocytes harbor HIV DNA in the brains of HIV-1-infected aviremic individuals on suppressive antiretroviral therapy. J Neuroimmune Pharmacol 14:110–119. 30194646 10.1007/s11481-018-9809-2 PMC6391194

[B49] Lee AM Miksys S Palmour R Tyndale RF (2006) CYP2B6 is expressed in African green monkey brain and is induced by chronic nicotine treatment. Neuropharmacology 50:441–450. 16309716 10.1016/j.neuropharm.2005.10.003

[B50] Lundgren DH Hwang SI Wu L Han DK (2010) Role of spectral counting in quantitative proteomics. Expert Rev Proteomics 7:39–53. 20121475 10.1586/epr.09.69

[B51] Ma Q Vaida F Wong J Sanders CA Kao YT Croteau D Clifford DB Collier AC Gelman BB Marra CM, ; CHARTER Group (2016) Long-term efavirenz use is associated with worse neurocognitive functioning in HIV-infected patients. J Neurovirol 22:170–178. 26407716 10.1007/s13365-015-0382-7 PMC4783211

[B52] Mann A Miksys SL Gaedigk A Kish SJ Mash DC Tyndale RF (2012) The neuroprotective enzyme CYP2D6 increases in the brain with age and is lower in Parkinson’s disease patients. Neurobiol Aging 33:2160–2171. 21958961 10.1016/j.neurobiolaging.2011.08.014

[B53] Mast N Verwilst P Wilkey CJ Guengerich FP Pikuleva IA (2020) In vitro activation of cytochrome P450 46A1 (CYP46A1) by efavirenz-related compounds. J Med Chem 63:6477–6488. 31617715 10.1021/acs.jmedchem.9b01383 PMC7226586

[B54] McFadyen MCE Melvin WT Murray GI (1998) Regional distribution of individual forms of cytochrome P450 mRNA in normal adult human brain. Biochem Pharmacol 55:825–830. 9586955 10.1016/s0006-2952(97)00516-9

[B55] Miksys S Lerman C Shields PG Mash DC Tyndale RF (2003) Smoking, alcoholism and genetic polymorphisms alter CYP2B6 levels in human brain. Neuropharmacology 45:122–132. 12814665 10.1016/s0028-3908(03)00136-9

[B56] Miksys S Rao Y Hoffmann E Mash DC Tyndale RF (2002) Regional and cellular expression of CYP2D6 in human brain: higher levels in alcoholics. J Neurochem 82:1376–1387. 12354285 10.1046/j.1471-4159.2002.01069.x

[B57] Mutlib AE Chen H Nemeth GA Markwalder JA Seitz SP Gan LS Christ DD (1999) Identification and characterization of efavirenz metabolites by liquid chromatography/mass spectrometry and high field NMR: species differences in the metabolism of efavirenz. Drug Metab Dispos 27:1319–1333. 10534318

[B58] Nightingale S Chau TT Fisher M Nelson M Winston A Else L Carr DF Taylor S Ustianowski A Back D, (2016) Efavirenz and metabolites in cerebrospinal fluid: relationship with CYP2B6 c.516G→T genotype and perturbed blood-brain barrier due to tuberculous meningitis. Antimicrob Agents Chemother 60:4511–4518. 27161633 10.1128/AAC.00280-16 PMC4958147

[B59] Ohno S Nakajin S (2009) Determination of mRNA expression of human UDP-glucuronosyltransferases and application for localization in various human tissues by real-time reverse transcriptase-polymerase chain reaction. Drug Metab Dispos 37:32–40. 18838504 10.1124/dmd.108.023598

[B60] Ouzzine M Gulberti S Ramalanjaona N Magdalou J Fournel-Gigleux S (2014) The UDP-glucuronosyltransferases of the blood-brain barrier: their role in drug metabolism and detoxication. Front Cell Neurosci 8:349. 25389387 10.3389/fncel.2014.00349 PMC4211562

[B61] Peluso MJ Ferretti F Peterson J Lee E Fuchs D Boschini A Gisslén M Angoff N Price RW Cinque P, (2012) Cerebrospinal fluid HIV escape associated with progressive neurologic dysfunction in patients on antiretroviral therapy with well controlled plasma viral load. AIDS 26:1765–1774. 22614889 10.1097/QAD.0b013e328355e6b2 PMC3881435

[B62] Petrov AM Lam M Mast N Moon J Li Y Maxfield E Pikuleva IA (2019) CYP46A1 activation by efavirenz leads to behavioral improvement without significant changes in amyloid plaque load in the brain of 5XFAD mice. Neurotherapeutics 16:710–724. 31062296 10.1007/s13311-019-00737-0 PMC6694340

[B63] Purnell PR Fox HS (2014) Efavirenz induces neuronal autophagy and mitochondrial alterations. J Pharmacol Exp Ther 351:250–258. 25161171 10.1124/jpet.114.217869 PMC4201278

[B64] Renaud HJ Cui JY Khan M Klaassen CD (2011) Tissue distribution and gender-divergent expression of 78 cytochrome P450 mRNAs in mice. Toxicol Sci 124:261–277. 21920951 10.1093/toxsci/kfr240 PMC3216415

[B65] Romão PR Lemos JC Moreira J de Chaves G Moretti M Castro AA Andrade VM Boeck CR Quevedo J Gavioli EC (2011) Anti-HIV drugs nevirapine and efavirenz affect anxiety-related behavior and cognitive performance in mice. Neurotox Res 19:73–80. 20012242 10.1007/s12640-009-9141-y

[B66] Seneviratne HK Hamlin AN Heck CJS Bumpus NN (2020) Spatial distribution profiles of emtricitabine, tenofovir, efavirenz, and rilpivirine in murine tissues following *in vivo* dosing correlate with their safety profiles in humans. ACS Pharmacol Transl Sci 3:655–665. 32832868 10.1021/acsptsci.0c00015 PMC7433710

[B67] Srinivas N Joseph SB Robertson K Kincer LP Menezes P Adamson L Schauer AP Blake KH White N Sykes C, (2019) Predicting efavirenz concentrations in the brain tissue of HIV-infected individuals and exploring their relationship to neurocognitive impairment. Clin Transl Sci 12:302–311. 30675981 10.1111/cts.12620 PMC6510381

[B68] Stamou M Wu X Kania-Korwel I Lehmler HJ Lein PJ (2014) Cytochrome p450 mRNA expression in the rodent brain: species-, sex-, and region-dependent differences. Drug Metab Dispos 42:239–244. 24255117 10.1124/dmd.113.054239 PMC3912540

[B69] Stapleton G Steel M Richardson M Mason JO Rose KA Morris RG Lathe R (1995) A novel cytochrome P450 expressed primarily in brain. J Biol Chem 270:29739–29745. 8530364 10.1074/jbc.270.50.29739

[B70] Stark K Wu ZL Bartleson CJ Guengerich FP (2008) mRNA distribution and heterologous expression of orphan cytochrome P450 20A1. Drug Metab Dispos 36:1930–1937. 18541694 10.1124/dmd.108.022020 PMC4694639

[B71] Suckow RF Smith TM Perumal AS Cooper TB (1986) Pharmacokinetics of bupropion and metabolites in plasma and brain of rats, mice, and guinea pigs. Drug Metab Dispos 14:692–697. 2877828

[B72] Tashima KT Caliendo AM Ahmad M Gormley JM Fiske WD Brennan JM Flanigan TP (1999) Cerebrospinal fluid human immunodeficiency virus type 1 (HIV-1) suppression and efavirenz drug concentrations in HIV-1-infected patients receiving combination therapy. J Infect Dis 180:862–864. 10438381 10.1086/314945

[B73] Thompson CG Bokhart MT Sykes C Adamson L Fedoriw Y Luciw PA Muddiman DC Kashuba AD Rosen EP (2015) Mass spectrometry imaging reveals heterogeneous efavirenz distribution within putative HIV reservoirs. Antimicrob Agents Chemother 59:2944–2948. 25733502 10.1128/AAC.04952-14 PMC4394831

[B74] Tovar-y-Romo LB Bumpus NN Pomerantz D Avery LB Sacktor N McArthur JC Haughey NJ (2012) Dendritic spine injury induced by the 8-hydroxy metabolite of efavirenz. J Pharmacol Exp Ther 343:696–703. 22984227 10.1124/jpet.112.195701 PMC3500535

[B75] Uchihashi S Nishikawa M Sakaki T Ikushiro S (2013) Comparison of serotonin glucuronidation activity of UDP-glucuronosyltransferase 1a6a (Ugt1a6a) and Ugt1a6b: evidence for the preferential expression of Ugt1a6a in the mouse brain. Drug Metab Pharmacokinet 28:260–264. 23089803 10.2133/dmpk.dmpk-12-nt-091

[B76] Uno Y Yamazaki H (2020a) Molecular characterization of UDP-glucuronosyltransferases 3A and 8A in cynomolgus macaques. Drug Metab Pharmacokinet 35:397–400. 32646660 10.1016/j.dmpk.2020.05.001

[B77] Uno Y Yamazaki H (2020b) mRNA levels of drug-metabolizing enzymes in 11 brain regions of cynomolgus macaques. Drug Metab Pharmacokinet 35:248–252. 31964621 10.1016/j.dmpk.2019.12.003

[B78] Upadhya SC Tirumalai PS Boyd MR Mori T Ravindranath V (2000) Cytochrome P4502E (CYP2E) in brain: constitutive expression, induction by ethanol and localization by fluorescence in situ hybridization. Arch Biochem Biophys 373:23–34. 10620320 10.1006/abbi.1999.1477

[B79] Uutela P Reinilä R Harju K Piepponen P Ketola RA Kostiainen R (2009) Analysis of intact glucuronides and sulfates of serotonin, dopamine, and their phase I metabolites in rat brain microdialysates by liquid chromatography-tandem mass spectrometry. Anal Chem 81:8417–8425. 19772284 10.1021/ac901320z

[B80] van Marle G Henry S Todoruk T Sullivan A Silva C Rourke SB Holden J McArthur JC Gill MJ Power C (2004) Human immunodeficiency virus type 1 Nef protein mediates neural cell death: a neurotoxic role for IP-10. Virology 329:302–318. 15518810 10.1016/j.virol.2004.08.024

[B81] van Rensburg R Nightingale S Brey N Albertyn CH Kellermann TA Taljaard JJ Esterhuizen TM Sinxadi PZ Decloedt EH (2022) Pharmacogenetics of the late-onset efavirenz neurotoxicity syndrome (LENS). Clin Infect Dis 75:399–405. 34882770 10.1093/cid/ciab961

[B82] Vera JH Bracchi M Alagaratnam J Lwanga J Fox J Winston A Boffito M Nelson M (2019) Improved central nervous system symptoms in people with HIV without objective neuropsychiatric complaints switching from efavirenz to rilpivirine containing cART. Brain Sci 9:195. 31405046 10.3390/brainsci9080195 PMC6721293

[B83] Voirol P Jonzier-Perey M Porchet F Reymond MJ Janzer RC Bouras C Strobel HW Kosel M Eap CB Baumann P (2000) Cytochrome P-450 activities in human and rat brain microsomes. Brain Res 855:235–243. 10677595 10.1016/s0006-8993(99)02354-9

[B84] Wahlström A Winblad B Bixo M Rane A (1988) Human brain metabolism of morphine and naloxone. Pain 35:121–127. 3237426 10.1016/0304-3959(88)90219-9

[B85] Walsh JG Reinke SN Mamik MK McKenzie BA Maingat F Branton WG Broadhurst DI Power C (2014) Rapid inflammasome activation in microglia contributes to brain disease in HIV/AIDS. Retrovirology 11:35. 24886384 10.1186/1742-4690-11-35 PMC4038111

[B86] Wang B Yang LP Zhang XZ Huang SQ Bartlam M Zhou SF (2009) New insights into the structural characteristics and functional relevance of the human cytochrome P450 2D6 enzyme. Drug Metab Rev 41:573–643. 19645588 10.1080/03602530903118729

[B87] Wang Y Liu M Lu Q Farrell M Lappin JM Shi J Lu L Bao Y (2020) Global prevalence and burden of HIV-associated neurocognitive disorder: a meta-analysis. Neurology 95:e2610–e2621. 32887786 10.1212/WNL.0000000000010752

[B88] Ward BA Gorski JC Jones DR Hall SD Flockhart DA Desta Z (2003) The cytochrome P450 2B6 (CYP2B6) is the main catalyst of efavirenz primary and secondary metabolism: implication for HIV/AIDS therapy and utility of efavirenz as a substrate marker of CYP2B6 catalytic activity. J Pharmacol Exp Ther 306:287–300. 12676886 10.1124/jpet.103.049601

[B89] World Health Organization (2018) Updated Recommendations on First-Line and Second-Line Antiretroviral Regimens and Post-Exposure Prophylaxis and Recommendations on Early Infant Diagnosis of HIV: Interim Guidelines. Supplement to the 2016 Consolidated Guidelines on the Use of Antiretroviral Drugs for Treating and Preventing HIV Infection, World Health Organization, Geneva, Switzerland.

[B90] Xu R Feng X Xie X Zhang J Wu D Xu L (2012) HIV-1 Tat protein increases the permeability of brain endothelial cells by both inhibiting ccluding expression and cleaving ccluding via matrix metalloproteinase-9. Brain Res 1436:13–19.

[B91] Yamaori S Jiang R Maeda C Ogawa R Okazaki H Aramaki H Watanabe K (2017) Expression levels of 39 Cyp mRNAs in the mouse brain and neuroblastoma cell lines, C-1300N18 and NB2a – strong expression of Cyp1b1. Fundam Toxicol Sci 4:195–200 DOI: 10.2131/fts.4.195.

[B92] Yu AM Idle JR Byrd LG Krausz KW Küpfer A Gonzalez FJ (2003) Regeneration of serotonin from 5-methoxytryptamine by polymorphic human CYP2D6. Pharmacogenetics 13:173–181. 12618595 10.1097/01.fpc.0000054066.98065.7b

[B93] Yun CH Park HJ Kim SJ Kim HK (1998) Identification of cytochrome P450 1A1 in human brain. Biochem Biophys Res Commun 243:808–810. 9500998 10.1006/bbrc.1998.8171

[B94] Zhong Y Dong G Luo H Cao J Wang C Wu J Feng YQ Yue J (2012) Induction of brain CYP2E1 by chronic ethanol treatment and related oxidative stress in hippocampus, cerebellum, and brainstem. Toxicology 302:275–284. 22960445 10.1016/j.tox.2012.08.009

[B95] Zimatkin SM Pronko SP Vasiliou V Gonzalez FJ Deitrich RA (2006) Enzymatic mechanisms of ethanol oxidation in the brain. Alcohol Clin Exp Res 30:1500–1505. 16930212 10.1111/j.1530-0277.2006.00181.x

